# Ribavirin Contributes to Hepatitis C Virus Suppression by Augmenting pDC Activation and Type 1 IFN Production

**DOI:** 10.1371/journal.pone.0135232

**Published:** 2015-08-14

**Authors:** Yang Wang, David R McGivern, Liang Cheng, Guangming Li, Stanley M Lemon, Junqi Niu, Lishan Su, Natalia J Reszka-Blanco

**Affiliations:** 1 Department of Translational Medicine and Department of Hepatology, the First Hospital of Jilin University, Changchun, Jilin, China; 2 Lineberger Comprehensive Cancer Center, The University of North Carolina at Chapel Hill, Chapel Hill, North Carolina, United States of America; 3 Department of Microbiology and Immunology, The University of North Carolina at Chapel Hill, Chapel Hill, North Carolina, United States of America; 4 Division of Infectious Diseases, Department of Medicine, The University of North Carolina at Chapel Hill, Chapel Hill, North Carolina, United States of America; Temple University School of Medicine, UNITED STATES

## Abstract

Ribavirin is used as a component of combination therapies for the treatment of chronic hepatitis C virus (HCV) infection together with pegylated interferon and/or direct-acting antiviral drugs. Its mechanism of action, however, is not clear. Direct antiviral activity and immunomodulatory functions have been implicated. Plasmacytoid dendritic cells (pDCs) are the principal source of type 1 interferon during viral infection. The interaction of pDCs with HCV-infected hepatocytes is the subject of intense recent investigation, but the effect of ribavirin on pDC activation has not been evaluated. In this study we showed that ribavirin augments toll-like receptors 7 and 9-mediated IFNα/β expression from pDCs and up-regulated numerous interferon-stimulated genes. Using the H77S.3 HCV infection and replication system, we showed that ribavirin enhanced the ability of activated pDCs to inhibit HCV replication, correlated with elevated induction of IFNα. Our findings provide novel evidence that ribavirin contributes to HCV inhibition by augmenting pDCs-derived type 1 IFN production.

## Introduction

Ribavirin (RBV) has been used for the treatment of chronic hepatitis C for decades. RBV is ineffective as monotherapy for chronic HCV infection [1, 2] but can boost rates of sustained virological response (SVR) when used in combination with pegylated interferon alpha and/or direct-acting antiviral (DAA) drugs. More effective DAA combination therapies may reduce the need for RBV, thus eliminating some of the undesirable side-effects of RBV such as anemia. In developing countries, however, RBV is a low cost drug and will likely remain an important component of HCV therapy for years to come. Despite its widespread long-term use, the antiviral mechanisms of RBV action are not fully understood [3–5].

RBV is a guanosine nucleoside analogue which possesses antiviral activity against both RNA and DNA viruses [6]. It has been successfully used against respiratory syncytial virus (RSV) [7], influenza virus, Lassa fever virus and hantavirus [8, 9]. However, its major application is in the treatment of chronic hepatitis C. Several mechanisms have been proposed to account for the antiviral activity of RBV [10]. RBV incorporation into viral genomes can promote the accumulation of mutations in progeny viruses that ultimately reduce their fitness and viability (the “error catastrophe” hypothesis) [11]. RBV can inhibit the host enzyme inosine monophosphate dehydrogenase (IMPDH) limiting the pools of cellular guanosine-5'-triphosphate (GTP) available for viral RNA replication [12]. RBV may also inhibit the viral polymerase [13]. In addition to the direct antiviral activities, RBV has been proposed to have broad immunomodulatory functions [14–16]. RBV may potentiate the effect of interferons (IFNs) by increasing the induction of interferon-stimulated genes (ISGs). In vitro, RBV was shown to directly up regulate ISGs [14, 17], enhance phosphorylation of STAT1/3 and increase expression of MxA in hepatocytes [18]. In vivo data are diverse, showing either elevated [19], only modestly increased, or unchanged levels of ISG expression [20] in RBV treated patients.

The benefits of RBV inclusion in DAA-based, IFNα-free regiments confirmed that RBV function extends beyond augmenting IFNα signaling [21–23]. It is proposed that RBV improves the anti-HCV therapies by prevention of the virological relapse in both IFNα and DAA-based therapies [24–28] but the mechanism of this action remains unknown. The most recent analysis of gene expression in patients on DAA sofosbuvir plus RBV revealed that restoration of endogenous IFNα2 and decrease of aberrant expression of type II and III IFNs, their receptors and ISGs, correlates with achievement of SVR and prevents virus relapse [29].However, it is unclear which cell population is mainly responsible for restoration of endogenous type 1 IFN (IFN-I) and whether RBV participate in this process. In infected hepatocytes, HCV efficiently blocks IFN-I production by NS3-4A mediated cleavage of mitochondrial antiviral signaling protein (MAVS) and TIR–domain-containing adapter-inducting interferon β (TRIF) [30, 31]. Therefore, uninfected bystander hepatocytes or virus-stimulated non-parenchymal cells are proposed to be the source of IFN-I in infected liver [32]. Among those cells, pDCs [33] are the most abundant source of IFN-I [34, 35]. In vitro studies have shown that pDCs do not respond directly to HCV but cell-to-cell contact of infected hepatocytes with pDCs results in toll-like receptor 7 (TLR7)-mediated production of IFNα [33, 36, 37]. During antiviral therapy pDCs are exposed to both HCV-infected cells and RBV, therefore RBV can potentially influence the cytokine expression profile of pDCs in the environment of infected hepatocytes. The effect of RBV on pDC activation has not been evaluated.

The aim of this study was to examine the effect of RBV on IFNα induction in pDCs. We hypothesized that RBV influences pDC function and modulates the host response to HCV infection. Using the pDC-Gen2.2 cell line we found that TLR7 and TLR9 (TLR7/9)-mediated IFN-I responses were increased by RBV. The increase in total IFNα/β levels amplified the induction of several antiviral ISGs. Furthermore, RBV enhanced IFNα production from pDC-Gen2.2 co-cultured with infected hepatocytes. The pDC-Gen2.2-derived, IFNα was sufficient to reduce HCV replication as efficiently as exogenous IFN-I. RBV elevated IFNα/β and ISGs production to enhance HCV clearance.

## Materials and Methods

### Cells

pDC-Gen2.2 were grown on the MS-5 feeder cell line [36] and the non-adherent fraction of the culture was used for all experiments. The Gen2.2-MS-5 co-culture and Gen2.2-Huh7.5 co-culture were propagated in RPMI 1640 medium (Gibco) supplemented with 10% fetal calf serum, 100 U penicillin, 100 U streptomycin, 1μM L-glutamine, 1x non-essential amino acids and 1mM sodium pyruvate. The Huh7.5 cells were grown in Dulbecco’s modified Eagle’s medium (DMEM) (Sigma) supplemented with 10% fetal calf serum, 100 U penicillin, 100 U streptomycin and 1mM L-glutamine. Total peripheral blood mononuclear cells (PBMCs) were isolated from peripheral blood of healthy donors by Ficoll-Paque™ Plus (GE Healthcare) density gradient centrifugation and maintained in complete RPMI 1640 (Gibco). The blood was obtained from Gulf Coast Regional Blood Center, Houston, Texas, USA.

### HCV Infection System

H77S.3/GLuc2A is an infectious molecular clone of genotype 1a HCV [38], which expresses *Gaussia* luciferase (*GLuc*) as a fusion with its polyprotein. In this genome, the *GLuc* sequence, followed by a foot and mouth disease virus 2A autoprotease, is inserted in frame between the p7 and NS2 sequences of H77S.3.When this genome is transfected into Huh7.5 cells, it can replicate and express GLuc, which is secreted into the medium. GLuc activity in the medium provides an indirect but sensitive measure of intracellular viral RNA replication. Previous studies have demonstrated that GLuc activity is proportional to intracellular HCV RNA abundance [39, 40]. Plasmids encoding H77S.3/GLuc2A or the replication-defective H77SAAG/GLuc2A (containing a mutation in the active site of the viral RNA-dependent RNA polymerase) were transcribed in vitro and the resulting genomic RNAs used to electroporate permissive Huh7.5 hepatoma cells as described previously [39]. Electroporated cells were cultured for 4 days to allow HCV replication to reach a steady state before seeding to 96-well plates at 8x10^3^ per well for co-culture or drug treatment experiments. Replication of H77S.3/GLuc2A typically resulted in GLuc activity in medium that was 1000-fold higher than background levels observed from cells transfected with the replication-incompetent H77SAAG/GLuc2A (“Mock-infected”).

### Gen2.2-Huh7.5 Co-culture

HCV-transfected and mock-transfected Huh7.5 cells were trypsinized and resuspended in complete RPMI 1640. 8000 cells were plated in 96 well round bottom plates and co-cultured with 800 pDC-Gen2.2 (the Huh7.5: Gen2.2 ratio was 10: 1). Co-cultures were treated with 0.25μM CpGA (Invivogen, tlrl-2216) and/or 5μM RBV (Sigma, R9644) or left untreated and maintained in complete RPMI 1640 supplemented with 1μM vitamin E (Sigma, 258024). At day 1, 2 and 3 cell-culture supernatants were collected. HCV replication was measured by Luciferase assay and qRT-PCR. IFNα levels were measured by ELISA.

### pDC-Gen2.2 activation with TLR7/9 and TLR2/4 ligands

pDC-Gen2.2 cells were cultured at 1–2 10^6 cells/ml in complete RPMI 1640 on the MS-5 feeder cells. Before the experiment, the non-adherent fraction of the culture was harvested, pelleted at 1500 rpm and re-suspended in a fresh medium. 50,000 cells were plated in 96-well round bottom plates and stimulated with 0.25μM CpGA (Invivogen, tlrl-2216), 0.25μM CpGB (Invivogen, tlrl-2006), 3.75μg/ml R848 (Invivogen, tlrl-r848), 0.5μg/ml LPS (Invivogen, tlrl-b5lps) or were left unstimulated (Mock) in total volume of 150μl. The stimulation assay was performed with and without 5μM RBV (1221 ng/ml) (Sigma, R9644). At time points (indicated in figures), the culture supernatants were analyzed for IFNα by ELISA and cells were harvested for RNA isolation and qRT-PCR analysis of gene expression. Where total PBMCs were used, 50,000 cells were stimulated with 0.25μM CpGA +/- RBV and analyzed as above.

### Depletion of pDCs from PBMCs

PBMCs were isolated as described above and pDCs were positively selected from PBMCs using BDCA-4 (CD304) magnetic beads (Miltenyi Biotec) according to the manufacturer’s protocol. The cells were stained with anti-CD4, CD3, CD303 and CD123 antibodies and death cell marker. The frequencies of each populations were assessed by fluorescence-activated cell sorter (FACS) staining. In PMBCs used for analysis, the input level of pDCs was 0.49% of leukocytes. After depletion, the pDCs level was reduced to 0.08%. pDC-depleted PBMCs were maintained in complete RPMI 1640 and used for stimulation as described above.

### ELISA

ELISA kits for IFNα detection (Mabtech, 3425-1H-20) were used according to the manufacturer’s instructions. The limit of ELISA detection was 10 pg/ml as indicated by manufacturer and confirmed in our laboratory by measuring the absorbance of serially diluted IFN-I standard, provided with the ELISA kit.

### Statistics

Statistical analyses were performed using Graphpad Prism software (GraphPad Software, Inc, La Jolla, CA, USA). Values are shown as mean with standard deviation (SD) or mean with standard error of mean (SEM) as indicated in figure legends. Each condition was performed in triplicate or as indicated in figure legends. One-way analysis of variance (ANOVA), followed by Bonferroni’s multiple-comparison test was used. * p≤0.05;**p≤0.01;***p≤0.001.

### Quantitative (q) RT-PCR

For the each tested condition the stimulation of pDC-Gen2.2 and PBMCs was performed in triplicate. RNA was isolated using RNeasy Mini kit (Qiagen, 74106). RNA was quantified using a Nanodrop spectrophotometer. 1–5μg of total RNA was used to make cDNA using the SSIII RT system (Invitrogen, 18080–051). Each reaction was performed in triplicate. qRT-PCR was performed using SYBR Green primers and master mix (Qiagen) and on a Quant Studio 6 Flex Real Time PCR System (Applied Biosystems/Life Technology), with the following conditions: 40 cycles of: 95°C for 15 sec; 58°C for 30 sec; 72° for 30 sec. Gene expression was normalized to GAPDH levels. Data was analyzed by the 2^-ΔΔCT^ method [41]. Primers used in the qRT-PCR assays were synthesized at Tissue Culture Facility, Nucleic Acid Division, LCCC, UNC. Primers for IFNα and IFNβ [33], IFNγ [42], PKR [18], TRIM22 [43] were described previously. Primers for type III IFN were as follows: IL-29-F: 5′CACATTGGCAGGTTCAAATCTCT-3′; IL-29-R: 5′-CCAGCGGACTCCTTTTTGG-3′; IL-28B-F:5′TAAGAGGGCCAAAGATGCCTT-3′; IL-28B-R:5′-CTGGTCCAAGACATCCCCC-3′. Primers for remaining genes analyzed in the study: OAS-1-F: 5′-TGTCCAAGGTGGTAAAGGGTG-3′; OAS-1-R:5′-CCGGCGATTTAACTGATCCTG-3′;STAT1-F: 5-′CAGCTTGACTCAAAATTCCTGGA-3′; STAT1-R: 5′-TGAAGATTACGCTTGCTTTTCCT-3′; IRF-9-F: 5′-GCCCTACAAGGTGTATCAGTTG-3′; IRF-9-R: 5′-TGCTGTCGCTTTGATGGTACT-3′.For HCV RNA quantification, the cells from co-cultures were harvested at day 3. Total RNA was extracted from the cells and quantified as above. HCV RNA was quantified by qRT-PCR as described previously [39].

### Cell Viability Assay

To assess the viability of pDC-Gen2.2 and Huh7.5 at different dose of RBV, the XTT Cell Viability Assay (Cell Signaling, 9095) was performed according to manufacturer’s instruction.

## Results

### RBV augments TLR7/9-mediated IFNα production in pDC-Gen2.2

To examine the role of RBV in pDCs activation we used the pDC cell line: pDC-Gen2.2 [44, 45]. pDC-Gen2.2 cells express classic markers and pathogen associated molecular patterns (PAMPs) of pDCs and have been shown to be a model cell line to study HCV pathobiology in the context of virus-cell interaction and IFN-I and III production [46]. To mimic pathogen-mediated stimulation of pDCs we used ligands: R848 and CpGA/B that are recognized by TLR7 and 9 respectively, to efficiently induce cytokine production in pDCs.

**Fig 1:**
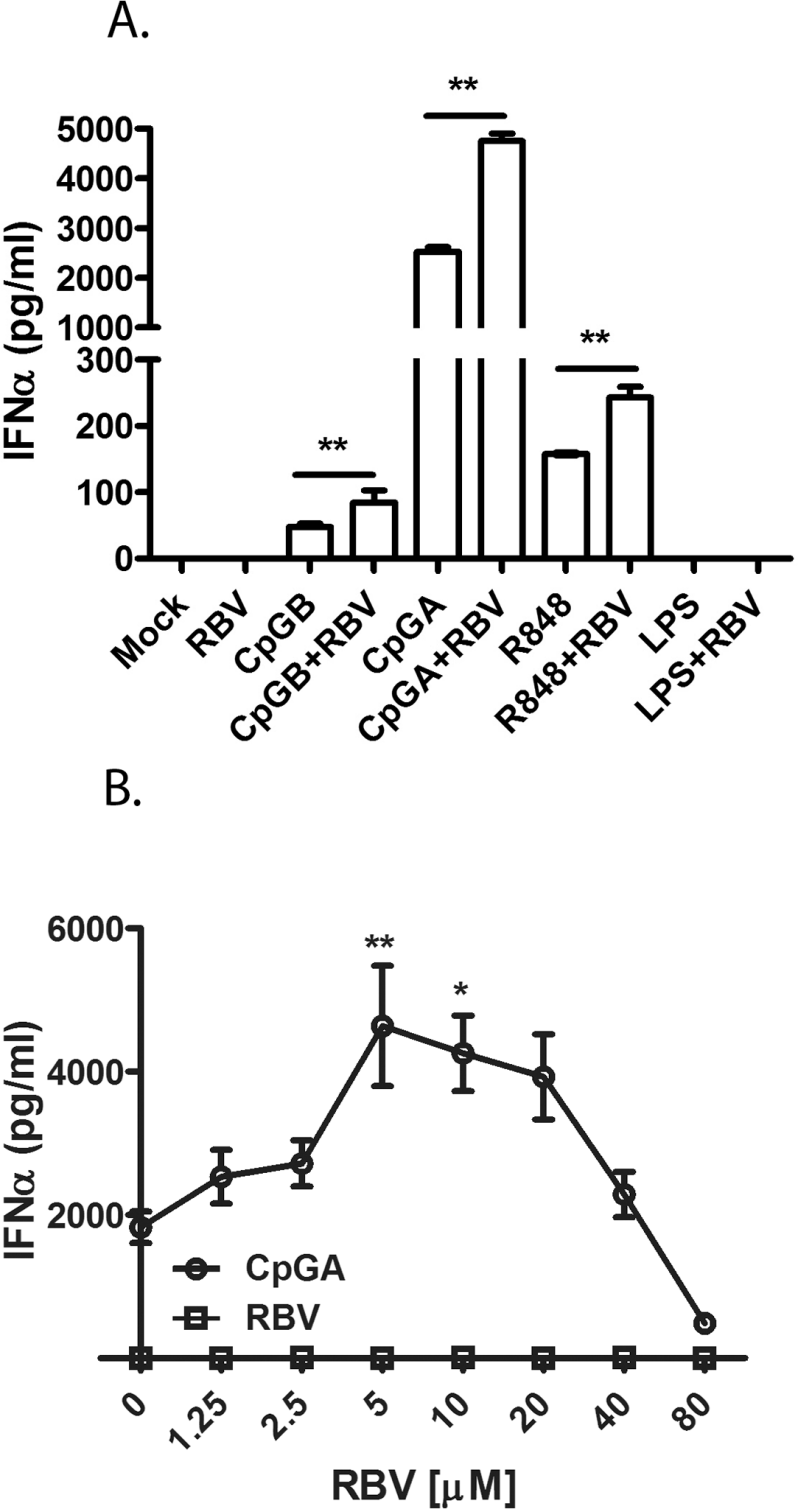
RBV augments TLR7/9-mediated pDC activation and IFNα induction. (A) RBV enhances IFNα production in pDC-Gen2.2 cells stimulated with CpGA, CpGB (TLR9) and R848 (TLR7). pDC-Gen2.2 were stimulated with TLR7/9 ligands in the presence and absence of RBV. Stimulation of pDC-Gen2.2 with LPS+/-RBV represents a negative control to show specificity. The values are shown as mean with SD. (B) Dose dependent effect of RBV on CpGA-induced IFNα production. pDC-Gen2.2 were stimulated with 0.25μM CpGA and with a range of RBV concentrations (from 1.25 to 80μM). IFNα was measured by ELISA at 16h post treatment (hpt). The values are shown as mean from four independent experiments with SEM. One-way analysis of variance (ANOVA), followed by Bonferroni’s multiple-comparison test was used to compare between treatment groups.**p≤0.01.

All ligands activated pDC-Gen2.2 to produce IFNα (Fig 1A). CpGA, a strong stimulant of TLR9 (S1A Fig), induced the highest level of IFNα (2521.84pg/ml). CpGB and R848, which are relatively weak activators of IFNα (S1A and S1B Fig), induced lower levels of IFNα than CpGA (48.05 pg/ml and 158.08 pg/ml, respectively). Interestingly, for each tested ligands, the co-stimulation with RBV resulted in two-fold higher level of IFNα than stimulation with ligand alone. The greatest induction of IFNα was observed for a combination of RBV and CpGA (4748.00 pg/ml). When applied alone, RBV was not able to activate pDC-Gen2.2. In contrast, the stimulation with LPS +/- RBV did not induce IFNα showing that enhancement of TLR7/9- mediated IFNα production by RBV is a specific effect.

Next, we estimated the optimal concentration of RBV necessary to augment ligand-mediated stimulation of pDC-Gen2.2 (Fig 1B). RBV enhancement of IFNα production was dose dependent. The drug increased the production of IFNα within the concentration range of 1.25μM-20μM, with peak saturation at 5μM. That concentration range was equivalent to the plasma concentration of RBV which correlates with achieving SVR [47–50]. RBV concentrations above 40μM reduced the IFNα production compared to stimulation with CpGA alone, most likely because CpGA and high dose RBV additive effects caused receptor oversaturation and reduced the viability of pDC-Gen2.2 (S4A Fig).

**Fig 2:**
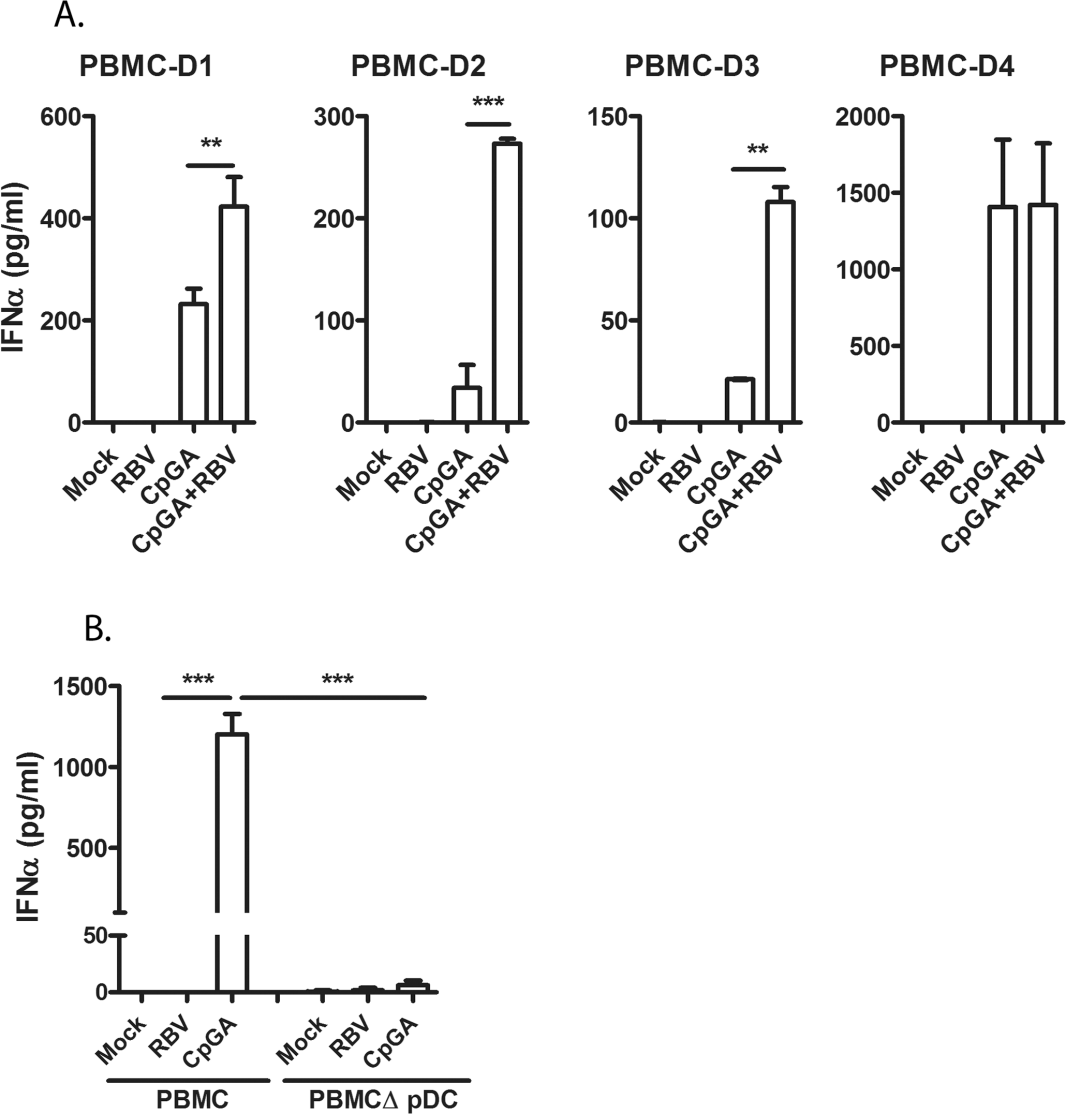
RBV enhancement of IFNα production in human PBMCs. (A) Effect of RBV on IFNα production from PBMCs of different donors. Total PBMCs were stimulated with CpGA in the presence and absence of RBV. IFNα was measured by ELISA at 12hpt. (B) Depletion of pDCs from PBMC significantly reduces CpGA mediated IFNα induction. Total PBMC and pDC- depleted PBMC (PBMCΔpDC) were stimulated with CpGA. IFNα was measured as above. The values are shown as mean with SD. One-way analysis of variance (ANOVA), followed by Bonferroni’s multiple-comparison test was used to compare between treatment groups. **p≤0.01; ***p≤0.001.

RBV enhancement of IFNα production was reproduced in total PBMCs stimulated with CpGA. PBMCs from eight donors were tested. The responsiveness to CpGA and to CpGA+RBV stimulation was diverse between PBMCs from each donor. To show the rate of change, the results of stimulation of PBMCs from four individuals are presented (Fig 2A). The PBMCs isolated from remaining four donors responded to stimulation with similar pattern. In most of the tested PMBCs, the RBV consistently enhanced the CpGA-mediated IFNα production by 2–8 fold. However, in PBMCs from certain donors, the changes in IFNα levels between CpGA and CpG+RBV stimulation were not observed. Interestingly, PBMCs of those donors responded to CpGA stimulation with relatively high level of IFNα. In average, the RBV treatment increased the IFNα production by 2 fold, consistent with the extent of enhancement observed in pDC-Gen2.2. The depletion of pDCs from PBMCs (S2 Fig) reduced the responsiveness to CpGA, proving that early IFNα induction *via* CpG-TLR9 pathways was entirely dependent on pDCs (Fig 2B).

### Expression of interferon-stimulated genes in CpGA and RBV treated pDC-Gen2.2

**Fig 3:**
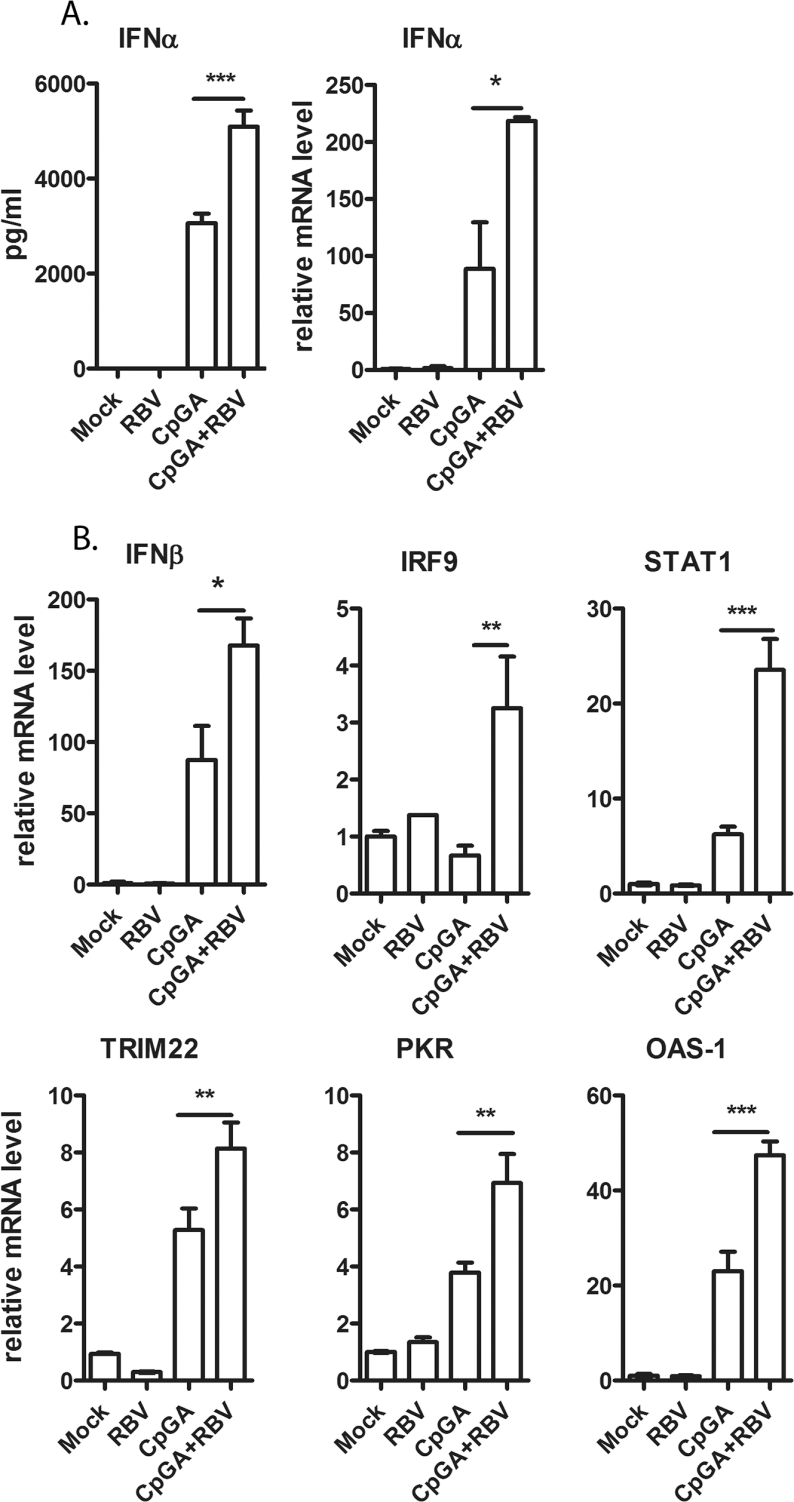
Expression of interferon-stimulated genes in CpGA and RBV treated pDC-Gen2.2. (A) Comparison of IFNα protein and mRNA levels in sample treated with CpGA and CpGA+RBV. Induction of IFNα was measured by ELISA or qRT-PCR, respectively at 16 hpt. (B) Induction of ISG expression in CpGA+/-RBV treated pDC-Gen2.2. ISG mRNA abundance was measured by qRT-PCR at 16hpt. The values are shown as mean with SD. One-way analysis of variance (ANOVA), followed by Bonferroni’s multiple-comparison test was used to compare between treatment groups. *p≤0.05; **p≤0.01; ***p≤0.001.

We were also interested to determine whether RBV augments induction of other types of IFNs besides IFNα, and/or enhances transcription of other genes which play a role in host resistance to viral infections. The elevated IFNα expression in the culture stimulated CpGA and RBV was confirmed by measuring both the secreted protein and cellular mRNA expression levels (Fig 3A). From the same samples, the induction of IFNβ and other ISGs were measured by quantitative RT-PCR (Fig 3B). Interestingly, combined CpGA and RBV treatment increased the level of IFNβ, but had no effect on expression of type II (IFNγ) and III (IL29-λ1 and IL28β-λ3) IFNs (data not shown). Moreover, RBV elevated levels of signal transducer and activator of transcription (STAT1) and interferon regulatory factor 9 (IRF9) transcription factors, which are directly involved in the IFNαβ receptor (IFNABR) JAK-STAT signaling pathway. As expected, the up-regulation of both IFNs resulted in elevated induction of ISGs in pDC-Gen2.2. RBV increased the expression of several critical components of the antiviral response like tripartite motif containing 22 (TRIM22), protein kinase R (PKR) and 2’-5’-oligoadenylate synthetase 1 (OAS-1).

### Kinetics of the RBV-enhanced IFNα expression in CpGA-activated pDCs

To gain insight into the kinetics of the RBV enhancement, we stimulated pDC-Gen2.2 with CpGA and RBV and monitored the level of IFNα production from 4h to 48h (S3 Fig). pDCs are known to robustly respond to CpGA stimulation within first 12h [51, 52] with significant amount of IFNα produced as early as 8h [53] after stimulation. Consistently, we did not observe any IFNα between 0-6h (data not shown). The secretion of IFNα started at 7hpt (hours post treatment) and increased within 24hpt. RBV gradually amplified the response by about two-fold starting from 7hpt up to 24hpt until the saturation observed at 48hpt. In sum, our results indicate that RBV augments the response to the TLR7, 9 ligand- mediated pDC-Gen2.2 stimulation and increases the level of IFNα production within the first 7hpt. We conclude that RBV acts at the early stage of IFNα production by mechanism that needs further elucidation.

### Ribavirin contributes to hepatitis C virus suppression by augmenting pDC activation and type 1 IFN production

**Fig 4:**
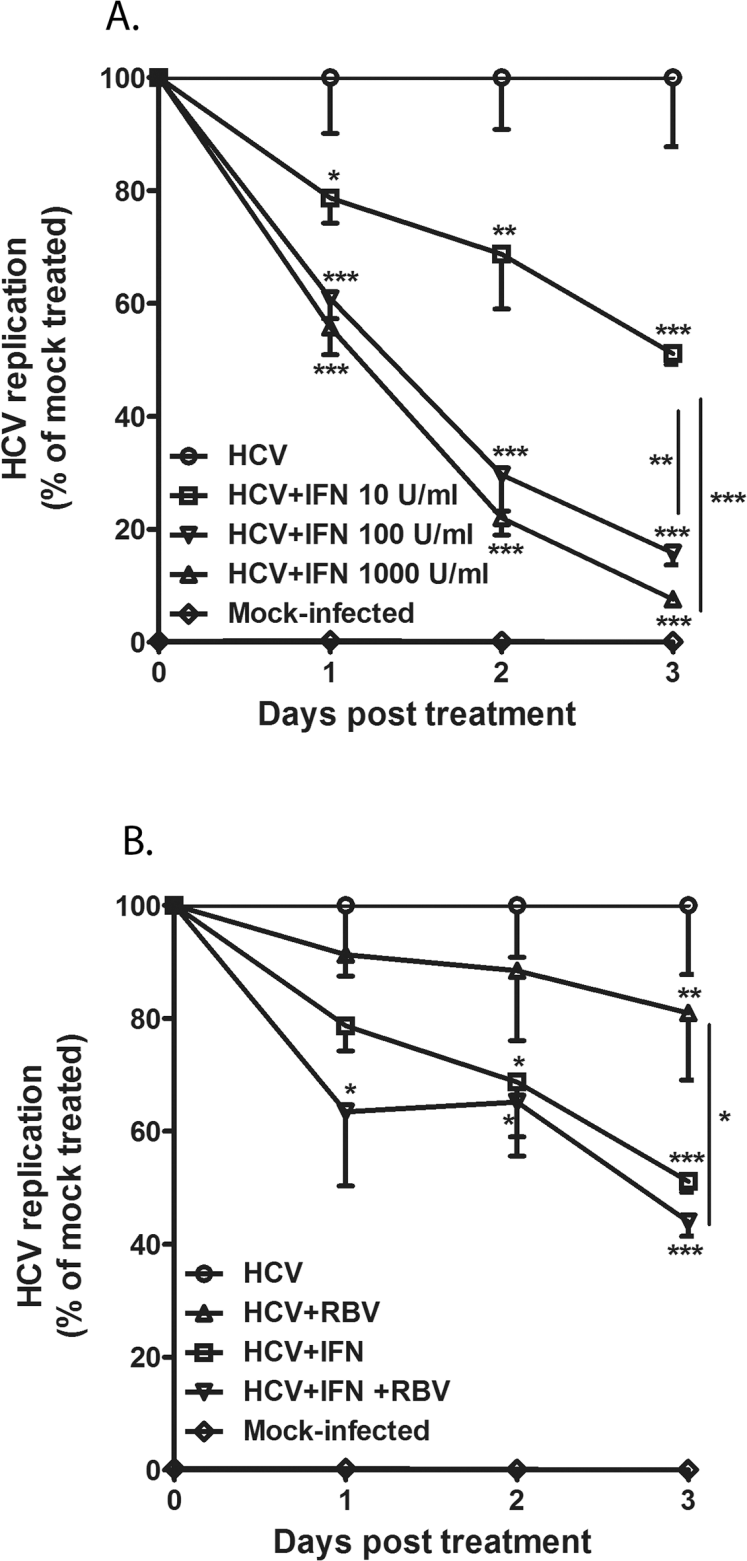
HCV sensitivity to exogenous IFN-I and RBV in H77S.3 Infection System. (A) IFN-I dose dependent control of HCV replication in the human hepatoma Huh7.5 cell line. HCV RNA-transfected Huh7.5 were grown to achieve stable replication and then cultured with different doses of recombinant IFN-I over 3 days. HCV replication was monitored by measurement of luciferase activity. (B) RBV effect on HCV replication in the H77S.3 Infection System. HCV RNA-transfected cells were grown in the presence of RBV and with the combination of low dose IFN-I (10u) and RBV. The viral replication was monitored as described above. The values are shown as mean with SD. One-way analysis of variance (ANOVA), followed by Bonferroni’s multiple-comparison test was used to compare between HCV and treatment groups and within the relevant groups as indicated in the figures. *p≤0.05, **p≤0.01,***p≤0.001.

First, we established the cell-culture model to study the sensitivity of HCV-infected hepatocytes to IFNα or RBV in order to study how RBV enhances pDC-Gen2.2-derived IFNα to inhibit HCV replication. We employed the H77S.3 infection system which allowed for robust HCV replication in the human hepatoma (Huh7.5) cells [38]. The viral replication in that reporter system was quantified by changes in secreted Gaussia luciferase (GLuc) activity [39]. HCV-transfected Huh7.5 were grown until stable HCV replication was achieved. To inhibit HCV replication, the infected hepatocytes were treated with increasing concentration of recombinant IFN-α (from 10 to 1000u/ml), and HCV replication of H77S.3 infection was monitored for 3 days (Fig 4A and S5 Fig). Addition of IFNα to the culture medium inhibited the viral replication in a dose-dependent fashion. The low dose of IFNα (10u/ml) inhibited HCV replication by 50%. The doses of 100 and 1000u/ml inhibited HCV replication to the same extent, which indicated that 100u/ml represents the sufficient concentration to inhibit HCV replication in the H77S.3 Infection System. Longer periods of IFN-treatment (up to day 6) did not further change the inhibition level as measured by luciferase activity (data not shown). RBV alone only marginally reduced HCV replication. However, differences between drug-treated and untreated groups showed significance at the day 3, what have suggested that longer period of time was required for direct antiviral effect of RBV in H77S.3 infection system (Fig 4B). When both RBV and IFNα were used, RBV showed no significant effect over IFNα-induced suppression of HCV replication (Fig 4B). RBV did not affect the viability of Huh7.5 as assessed by measuring the metabolic activity and viability of RBV treated hepatocytes (S4B Fig). Also, we did not observe any apparent changes in the proliferation between HCV-infected Huh7.5 treated with IFN (at the concentration of 100u/ml and 10u/ml +/-RBV) and cells from those groups reached confluence at the same time as the untreated HCV-infected Huh7.5.

**Fig 5:**
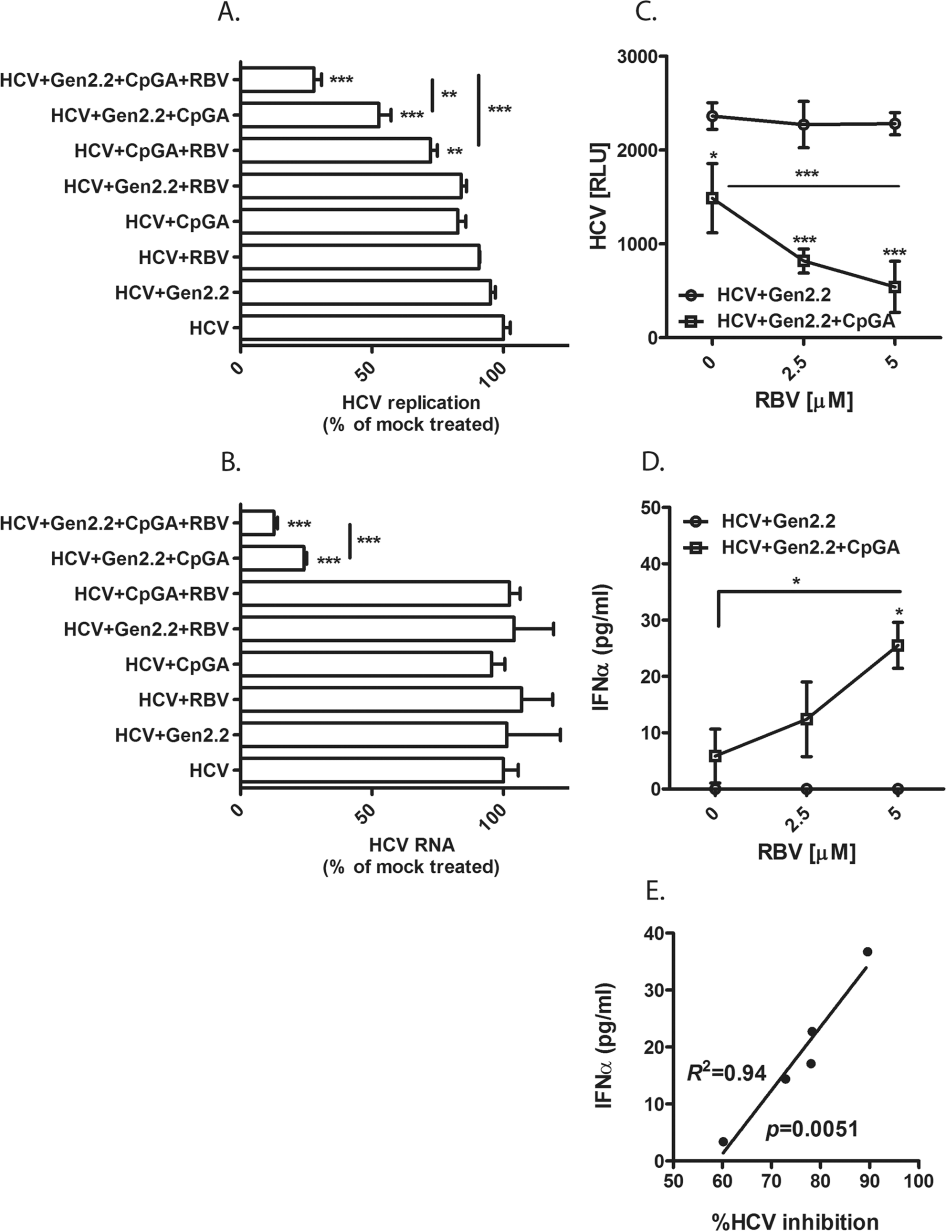
RBV enhances CpG-activated pDCs to produce IFN-I and inhibit HCV replication in H77S.3 cells. Huh7.5 cells stably infected with the reporter virus H77S.3/GLuc2A were co-cultured with pDC-Gen2.2 cells activated with CpGA or CpGA plus RBV for 3 days. The HCV replication was monitored by measurement of luciferase activity (A) and HCV RNA level by RT-PCR (B). Results are expressed as present of HCV-infected control. One-way analysis of variance (ANOVA), followed by Bonferroni’s multiple-comparison test was used to compare between HCV and treatment groups and within the relevant groups as indicated in the figures. (C) Inhibition of HCV replication by CpGA together with RBV correlates with (D) the induction of IFNα from pDC-Gen2.2. pDC-Gen2.2 were stimulated with CpGA and 2 different doses of RBV and co-cultured with HCV infected Huh7.5. The HCV replication was monitored by measurement of luciferase activity. Supernatants from co-cultures were harvested on day 3 to measure IFNα by ELISA. One-way analysis of variance (ANOVA), followed by Bonferroni’s multiple-comparison test was used to compare between treatment groups. *p≤0.05, **p≤0.01,***p≤0.001. The values are shown as mean with SD.(E) Correlation of pDC-derived IFNα and inhibition of HCV replication.

Our next focus was to determine the ability of pDC-Gen2.2-derived IFNα to inhibit HCV replication. Also, we asked if RBV enhanced pDC-Gen2.2 activation in the presence of infected hepatocytes and contributed to more efficient HCV inhibition. We used the H77S.3 Infection System to allow for HCV stable replication, then co-cultured the HCV infected hepatocytes with pDCs-Gen2.2 in the presence and absence of CpGA and/or RBV (Fig 5). The pDC-Gen2.2 were stimulated with CpGA+/-RBV or mock treated and added into the freshly seeded HCV-infected Huh7.5. Both cell types were in direct contact with each other. Within hours, activated pDC-Gen2.2 formed clusters, a typical sign of pDC-Gen2.2 activation. During the 3 day co-culture period, the HCV-infected Huh7.5 cells alone or in combination with RBV did not activate pDC-Gen2.2 to produce IFNα or inhibit HCV replication as measured by luciferase activity (Fig 5A) and HCV RNA level (Fig 5B). Culture of HCV-infected Huh7.5 with either CpGA alone or CpGA+RBV or pDC-Gen2.2+RBV only moderately reduced HCV replication. However, such small differences were observed only by measurement of luciferase activity, which represents a more sensitive assay for detection of small variations. IFNα was not detected in those samples (data not shown). Activation of pDC-Gen2.2 with CpGA significantly reduced the viral replication (Fig 5A and 5B). Remarkably, the simultaneous activation of pDC-Gen2.2 with CpGA and RBV potentiated the inhibition by 2–fold compared to CpG alone, as confirmed by measuring luciferase activity and HCV RNA level. Using these two readouts of virus RNA replication in independent experiments, we observed an identical trend of RBV-dependent enhancement of the antiviral effect in CpGA-stimulated co-cultures.

To determine whether reduction in viral replication was correlated with induction of IFNα, we monitored the viral growth and the level of pDC-Gen2.2–derived IFNα in co-cultures stimulated with CpGA and with two different concentrations of RBV (Fig 5C and 5D). CpGA+/-RBV induced IFNα production from pDC-Gen2.2 (Fig 5D) correlated with inhibition of HCV replication (Fig 5C and 5E). Therefore, co-stimulation with CpGA and RBV augmented the production of IFNα. We conclude, that in CpGA+RBV stimulated pDC-Gen2.2, the synergistic action of IFNα/β and ISGs induce a robust antiviral response resulting in inhibition of HCV replication in our co-culture model.

## Discussion

In this study we show that RBV is a modulator of pDCs activation. The activation of pDC-Gen2.2 by TLR7/9 ligands is enhanced by RBV, resulting in elevated IFN-I response. Increases in IFNα/β production upregulated numerous critical antiviral molecules, including PKR and OAS-1. In co-culture experiments with H77S.3 infected Huh7.5 cells, RBV enhanced the anti-HCV activity of pDC to inhibit HCV replication, correlated with levels of IFNα produced by activated pDC-Gen2.2. The study thus reveals a novel activity of RBV that contributes to HCV viral control by augmenting pDC activation and IFN-I production.

It has been proposed that the plasma concentration of RBV influences the achievement of SVR and can be a predictor of HCV relapse in IFN-based [47] and IFN-free therapies [26, 54]. Although RBV dose is weight-based, its plasma and tissue concentrations are variable also as a result of inter-individual conditions [55] and tissue-dependent uptake [56, 57]. RBV concentrations between 1000 to 4000ng/ml are detected in the serum of HCV-infected patients [49, 58, 59]. The end of treatment (EOT) concentration above 1,960ng/ml is reported to be predictive of SVR [47]. We show that the RBV concentration from 2.5μM to 20μM (610- 4884ng/ml) augments IFNα production from pDC-Gen2.2. This concentration range is below the toxicity threshold as measured for hepatocytes [17] (S4B Fig) and is equivalent to the range reported in plasma of patients achieving SVR. Consequently, RBV plasma concentrations that have positive treatment outcomes also correlate with concentrations that enhance TLR7/9-induced pDC activation and IFNα production. Our work showed that pDC-Gen2.2 cells tolerated this dose of RBV, with only marginal reduction in their metabolic activity of about 10% (S4A Fig). Importantly, RBV treatment up to 10μM does not have an effect on pDC-Gen2.2 stimulation ability and on IFN-I production (Figs 1A, 1B and 3).

TLR7/9–mediated induction of IFN-I involves engagement of the MyD88 adapter molecule and transcription factor IRF-7[60]. The secreted IFN-I can bind to the IFNABR receptor on pDC to trigger the JAK1-STAT pathway to promote expression of ISGs including IRF7 [61]. The induced IRF7 then initiates the expression of a second wave of IFN-I and ISGs. This autocrine-paracrine loop allows for massive production of IFN-I in response to stimulation [52, 53]. Our experiments show that RBV enhances IFNα production within 7 hours and suggest that RBV augments the CpGA-stimulated TLR7/9-IRF7signaling pathway rather than the IFN-I activated JAK1-STAT signaling pathway. It will be of future interest to elucidate the signaling pathway that is enhanced by RBV and dissect if RBV upregulate either TLR7/9-IRF7 or JAK1-STAT signaling pathways. Our results describe a novel function of RBV as a modulator of TLR7/9 signaling in pDCs. Following external stimulation with TLR7/9 ligand, pDC-Gen2.2 derived IFNα/β is augmented by RBV. The increases in total levels of IFN-I result in the induction of several antiviral genes that are hallmarks of the antiviral state.

To study the impact of pDCGen2.2 –derived IFNα induced by CpG and RBV on HCV replication, we used the H77S.3 Infection-Replication System [39]. Co-cultivation of H77S.3 infected Huh7.5 with pDC-Gen2.2 did not activate pDC-Gen2.2, as reported with primary pDCs co-cultured with HCV RNA-producing human hepatocytes [33, 37]. The difference may be due to differences in the cell lines and in the time period of co-culture. The H77S.3 infection model is less cytopathic than the JFH1 system [62] employed in previous studies [33].

In the set of our experiments RBV alone only minimally reduced HCV replication in the H77S.3 infection model. Although RBV alone may have no measurable antiviral effect in the relatively short time course that we have shown, it is possible that RBV may have a direct effect on virus replication over weeks or months of treatment (e.g. by promoting accumulation of mutations that negatively impact virus fitness–the error catastrophe hypothesis).

RBV has been added to IFN-based regimens since 1998 [63]. The benefit of RBV has been evaluated in multiple clinical trials [64–71] but its mechanism of action remains controversial. Since the approval of the first DAA (boceprevir and telaprevir) in 2011, many additional agents has been evaluated and approved in combination with or without RBV. The preferred treatment regimen depends on a variety of factors including patient disease state and HCV genotype. Currently, RBV combined with DAA is still recommended in patients with the majority of HCV genotypes (IDSA, http://hcvguidelines.org). Interestingly, recent evidence indicates that DAA therapy for 12 weeks can result in SVR even without RBV [68, 69], suggesting that removal of this drug from DAA-based therapies is possible. Understanding how RBV acts could allow its application only where necessary or to exchange it for a less toxic alternative. In the era of DAAs, the enigmatic mechanism of RBV-dependent SVR is an important matter that needs to be clarified to successfully control HCV infection. In this report, we show that RBV is a co-stimulator of TLR7/9 signaling pathway in pDCs and increases IFN-I-mediated inhibition of HCV replication. Further studies are needed to confirm the role of pDC-derived IFN-I in HCV clearance and the mechanism of RBV in the induction of IFN-I in human pDC.

## Supporting Information

S1 Fig Saturation of TLR7,9-mediated IFNα responses with increasing concentrations of the ligands. pDC-Gen2.2 were stimulated with different concentrations of TLR9 ligands: CpGA or CpGB (A) or with TLR7 ligand: R848 (B) and the IFNα was measured by ELISA at 18hpt. The values are shown as mean with SD.
(PDF)

S2 Fig The purity of pDC-deleted PBMCs assessed by FACS. The total human PBMCs and pDC-depleted PBMCs, were stained with anti-CD4, CD3, CD123 and CD303 antibodies and death cell marker. The percentage of CD123+, CD303+ cells in population of live CD3-CD4+ cells is presented.
(PDF)

S3 Fig The kinetics of RBV enhancement of CpGA-mediated IFNα induction. pDC-Gen2.2 were stimulated with CpGA in the presence and absence of RBV, the samples were harvested at 4hpt (data not shown), 7, 24 and 48 hpt to measure IFNα by ELISA. One-way analysis of variance (ANOVA), followed by Bonferroni’s multiple-comparison test was used to compare between treatment groups. The values are shown as mean with SD.*p≤0.05, **p≤0.01.
(PDF)

S4 Fig Viability of pDC-Gen22 and Huh7.5 cultured in the presence of RBV. pDC–Gen22 (A) and Huh7.5 (B) were treated with increasing concentration of RBV and the viability test was performed at 24, 48 and 96h after addition of the drug. Cell viability is presented as a percentage of mock- treated cells (100%). The values are shown as mean with SD.
(PDF)

S5 Fig Kinetics of HCV replication and sensitivity to exogenous IFN-I in H77S.3 Infection System. H77S.3-infected Huh7.5 cells were cultured with different doses of recombinant IFN-I over 3 days. The HCV replication was monitored by measurement of luciferase activity. The primary data of relative light units (RLU) are presented. One-way analysis of variance (ANOVA), followed by Bonferroni’s multiple-comparison test was used to compare between HCV (without IFN) and IFN treated groups and within the relevant groups as indicated in the figure. The values are shown as mean with SD. *p≤0.05, **p≤0.01,***p≤0.001.
(PDF)
