# Supplementary figures and images for: Ribavirin Contributes to Hepatitis C Virus Suppression by Augmenting pDC Activation and Type 1 IFN Production
Source: PLoS One. 2015 Aug 14;10(8):e0135232. doi: 10.1371/journal.pone.0135232 (PMC4537094; doi:10.1371/journal.pone.0135232)

S1 Figure

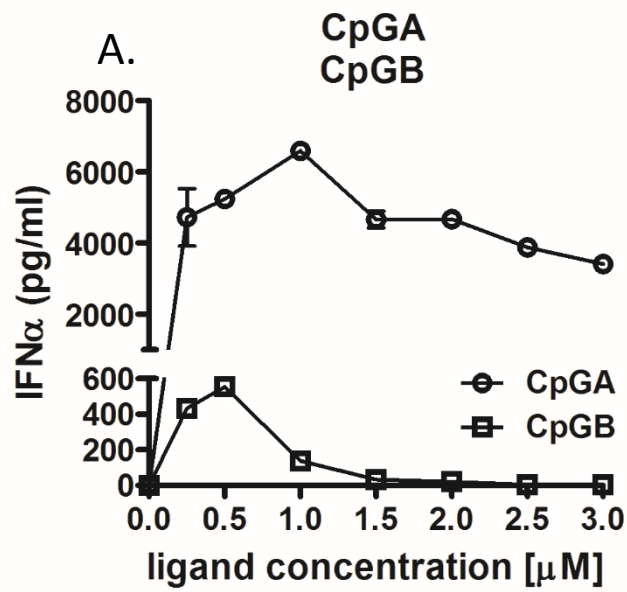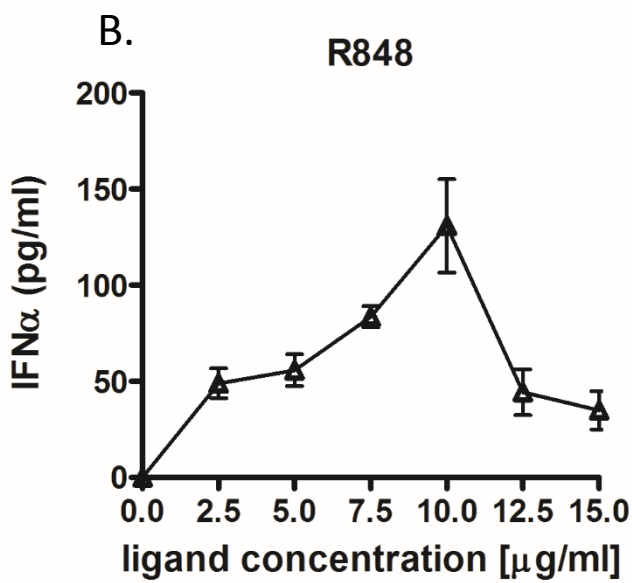

Supplement: S1 Fig — pDC-Gen2.2 were stimulated with different concentrations of TLR9 ligands: CpGA or CpGB (A) or with TLR7 ligand: R848 (B) and the IFNα was measured by ELISA at 18hpt. The values are shown as mean with SD. (PDF) [file pone.0135232.s001.pdf]

S2 Figure

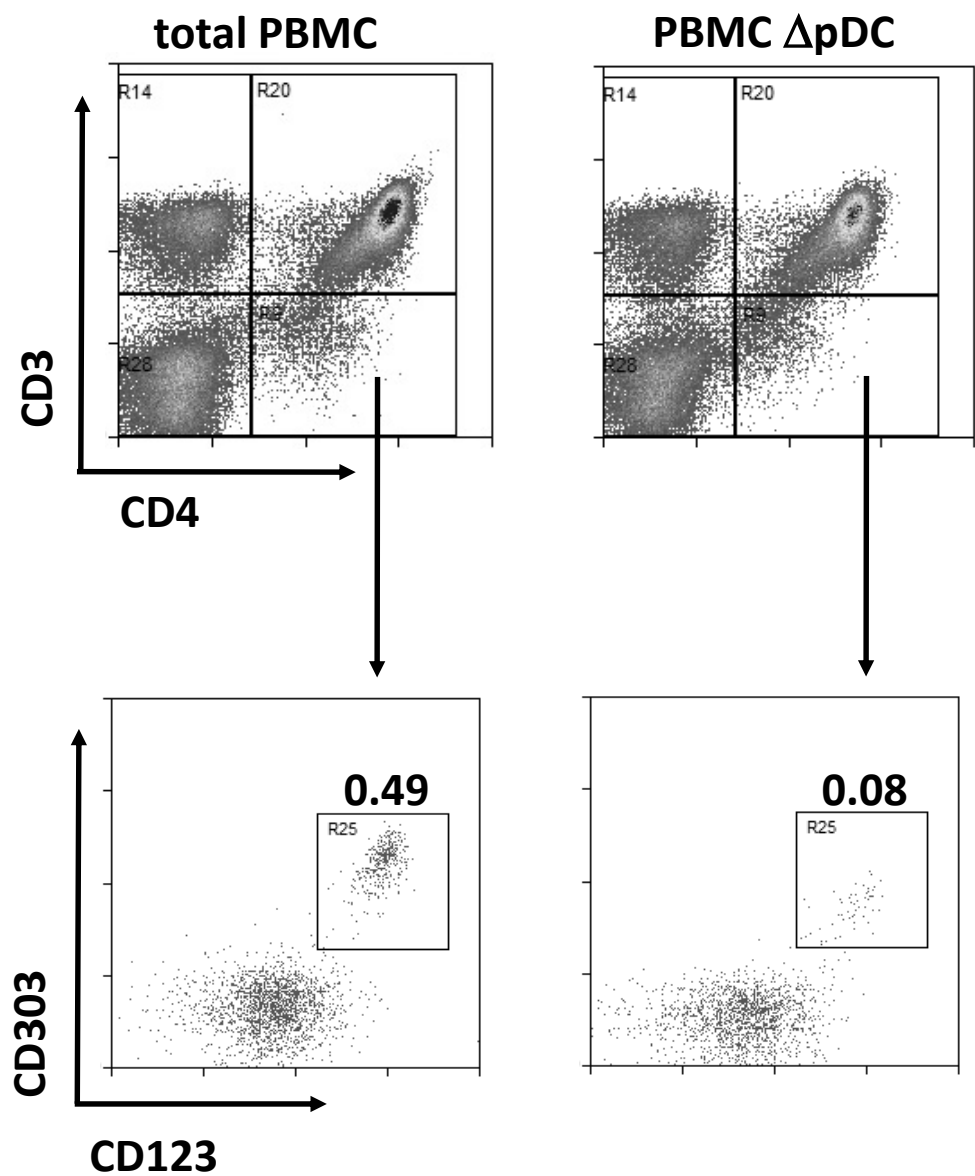

Supplement: S2 Fig — The total human PBMCs and pDC-depleted PBMCs, were stained with anti-CD4, CD3, CD123 and CD303 antibodies and death cell marker. The percentage of CD123+, CD303+ cells in population of live CD3-CD4+ cells is presented. (PDF) [file pone.0135232.s002.pdf]

S3 Figure

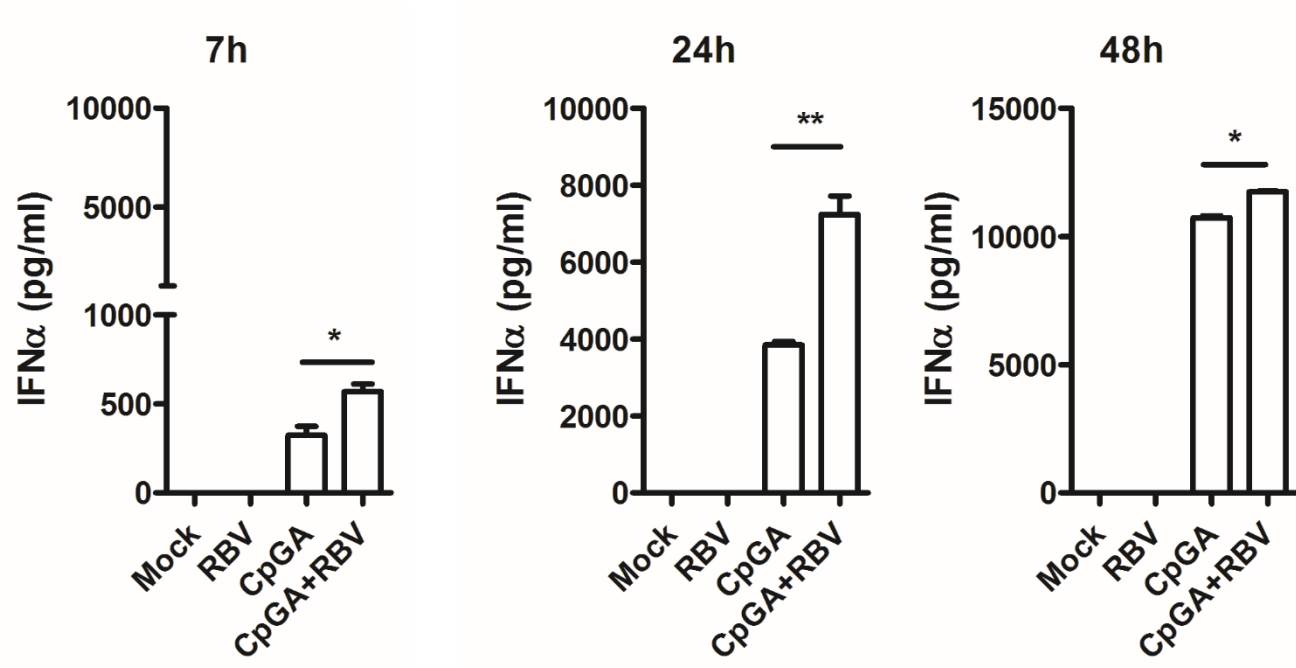

Supplement: S3 Fig — pDC-Gen2.2 were stimulated with CpGA in the presence and absence of RBV, the samples were harvested at 4hpt (data not shown), 7, 24 and 48 hpt to measure IFNα by ELISA. One-way analysis of variance (ANOVA), followed by Bonferroni’s multiple-comparison test was used to compare between treatment groups. The values are shown as mean with SD.*p≤0.05, **p≤0.01. (PDF) [file pone.0135232.s003.pdf]

S4 Figure

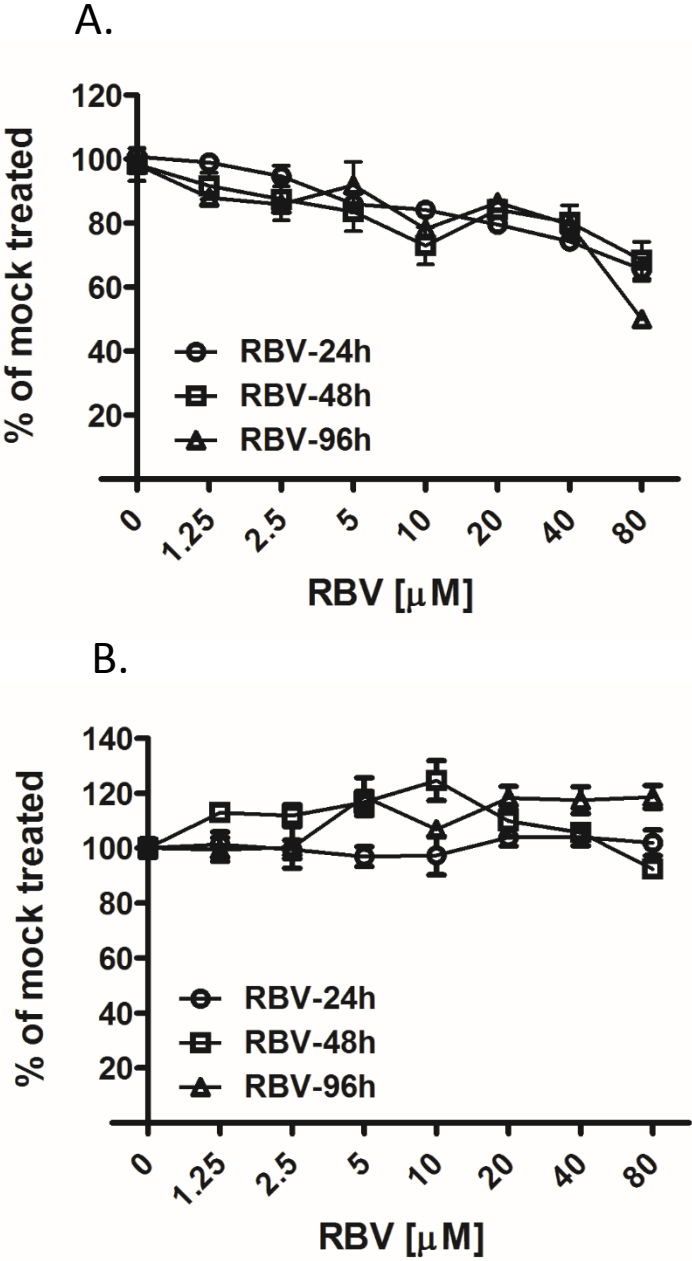

Supplement: S4 Fig — pDC–Gen22 (A) and Huh7.5 (B) were treated with increasing concentration of RBV and the viability test was performed at 24, 48 and 96h after addition of the drug. Cell viability is presented as a percentage of mock- treated cells (100%). The values are shown as mean with SD. (PDF) [file pone.0135232.s004.pdf]

S5 Figure

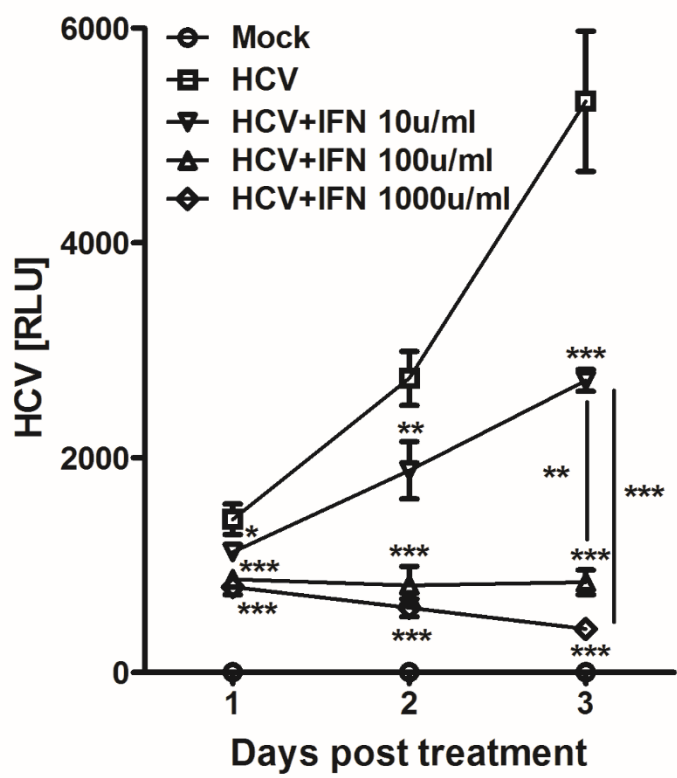

Supplement: S5 Fig — H77S.3-infected Huh7.5 cells were cultured with different doses of recombinant IFN-I over 3 days. The HCV replication was monitored by measurement of luciferase activity. The primary data of relative light units (RLU) are presented. One-way analysis of variance (ANOVA), followed by Bonferroni’s multiple-comparison test was used to compare between HCV (without IFN) and IFN treated groups and within the relevant groups as indicated in the figure. The values are shown as mean with SD. *p≤0.05, **p≤0.01,***p≤0.001. (PDF) [file pone.0135232.s005.pdf]
